# Diagnostic performance of ultrasonographic medial gastrocnemius muscle thickness for sarcopenia in patients on maintenance hemodialysis

**DOI:** 10.3389/fmed.2026.1926926

**Published:** 2026-09-03

**Authors:** Meilin Yan, Wei An, Leting Zhou, Weiwei Shan, Xiran Zhang, Aiyan Du, Zhijian Zhang, Liang Wang

**Affiliations:** Department of Nephrology, The Affiliated Wuxi People's Hospital of Nanjing Medical University, Wuxi People's Hospital, Wuxi Medical Center, Nanjing Medical University, Wuxi, Jiangsu, China

**Keywords:** bioelectrical impedance analysis, hemodialysis, medial gastrocnemius muscle thickness, muscle ultrasound, sarcopenia

## Abstract

**Objective:**

The study aimed to evaluate the performance of ultrasonographic medial gastrocnemius muscle thickness (MGMT) and pennation angle (MGMPA) in diagnosing sarcopenia among patients on maintenance hemodialysis (MHD).

**Methods:**

The MGMT and MGMPA were measured ultrasonographically. Baseline characteristics were compared between the sarcopenia and non-sarcopenia groups. Correlations between ultrasound parameters and sarcopenia indicators were evaluated. The diagnostic performance of the ultrasound parameters was assessed using receiver operating characteristic (ROC) curve analysis. Multivariable logistic regression analysis was conducted to identify independent predictors associated with sarcopenia, followed by the construction and validation of a diagnostic nomogram. Additionally, bootstrap mediation analysis assessed the direct and indirect effects of sonographic parameters on sarcopenia.

**Results:**

This study cohort comprised 221 MHD patients. Both MGMT (12.8 vs. 16.5 mm, *p* < 0.001) and MGMPA (26.35° vs. 29.73°, *p* < 0.001) were significantly reduced in the sarcopenia group compared to the non-sarcopenia group. MGMT exhibited significant positive correlations with calf circumference, appendicular skeletal muscle mass index (ASMI), appendicular skeletal muscle mass (ASM), body mass index (BMI), and handgrip strength, alongside a significant inverse correlation with the SARC-CalF score. MGMT demonstrated good diagnostic performance for sarcopenia, yielding an area under the curve (AUC) of 0.823 (*p* < 0.001). Multivariable logistic regression identified MGMT, BMI, older age, and male gender as independent predictors of sarcopenia. A predictive nomogram incorporating these factors demonstrated excellent discrimination (AUC = 0.873), good calibration, and potential clinical decision-making value across a wide threshold probability range. Mediation analysis confirmed a significant direct association between MGMT and the presence of sarcopenia in both the age model (ADE = −0.00303, *p* < 0.001) and the serum albumin model (ADE = −0.00251, *p* < 0.001), with no significant mediating effects observed for either variable.

**Conclusion:**

Ultrasonographic measurement of MGMT demonstrates high diagnostic performance and serves as a reliable, independent predictor for sarcopenia in patients undergoing maintenance hemodialysis, highlighting its potential as a practical, non-invasive screening tool in clinical practice.

## 1 Introduction

Sarcopenia is characterized by the progressive loss of skeletal muscle mass and strength. In the context of chronic kidney disease (CKD), particularly among patients undergoing maintenance hemodialysis (MHD), the prevalence of sarcopenia is notably high, ranging from 20% to 36.5% (1–3). This high burden is driven by a complex interplay of factors, including protein-energy wasting, chronic inflammation, metabolic disturbances, dialysis-induced catabolism, and oxidative stress. Clinical factors such as advanced age, low body mass index (BMI), and diabetes further increase the susceptibility (4), contributing to a higher risk profile within this vulnerable population. Given that sarcopenia is closely associated with reduced survival, intradialytic hypotension, adverse cardiovascular events, and elevated healthcare costs, timely and accessible screening remains clinically crucial (5–8). The diagnosis of sarcopenia typically requires a comprehensive assessment of muscle mass, muscle strength, and physical performance. Currently, the most widely recognized international diagnostic criteria are the consensus guidelines established by the European Working Group on Sarcopenia in Older People (EWGSOP) (9). However, considering the inherent differences in body size, lifestyle, and ethnicity between Western and Asian populations, the Asian Working Group for Sarcopenia (AWGS 2019) introduced specific diagnostic criteria and cut-off values tailored for Asian individuals (10). Given that our study population consists of Asian patients, the AWGS 2019 consensus was adopted as the primary diagnostic framework to ensure clinical relevance and accuracy. These guidelines recommend several techniques for evaluating muscle mass, including dual-energy X-ray absorptiometry (DXA), computed tomography (CT), magnetic resonance imaging (MRI), and bioelectrical impedance analysis (BIA). Among these, DXA and BIA are frequently utilized in clinical practice due to their diagnostic performance and feasibility. Although DXA and CT are less susceptible to body fluid fluctuations, their high cost, lack of portability, and associated radiation exposure often limit their utility for routine clinical screening (11). In contrast, the portability and bedside operability of BIA facilitate its widespread use; however, its accuracy in estimating muscle mass can be significantly confounded by the severe fluid shifts inherent to patients undergoing MHD.

Consequently, owing to its strong correlation with CT and its ability to evaluate individual muscles, there has been growing interest in muscle ultrasound as a versatile, non-invasive tool for assessing muscle mass and structure (12, 13). Currently, muscle thickness is increasingly utilized in the evaluation of sarcopenia, whereas the pennation angle is regarded as a potential indicator reflecting subtle structural alterations (14, 15). In this study, both medial gastrocnemius muscle thickness (MGMT) and medial gastrocnemius muscle pennation angle (MGMPA) were quantified using ultrasound in patients with MHD. Therefore, this study aimed to evaluate the diagnostic performance of MGMT and MGMPA for sarcopenia, thereby exploring their potential as accessible and routine screening tools in this vulnerable clinical population.

## 2 Subjects and methods

### 2.1 Subjects

This cross-sectional study was approved by the Ethics Committee of Wuxi People's Hospital (Approval No. KS202045) and conducted in accordance with the Declaration of Helsinki. All patients provided written informed consent prior to enrollment.

The sample size was calculated based on previously reported diagnostic accuracy data for ultrasonographic gastrocnemius muscle thickness (16, 17). Assuming a significance level (α) of 0.05, a margin of error of 0.10, and an estimated sarcopenia prevalence of 30% in this population, the minimum required sample size was determined to be approximately 210 participants.

**Figure 1:**
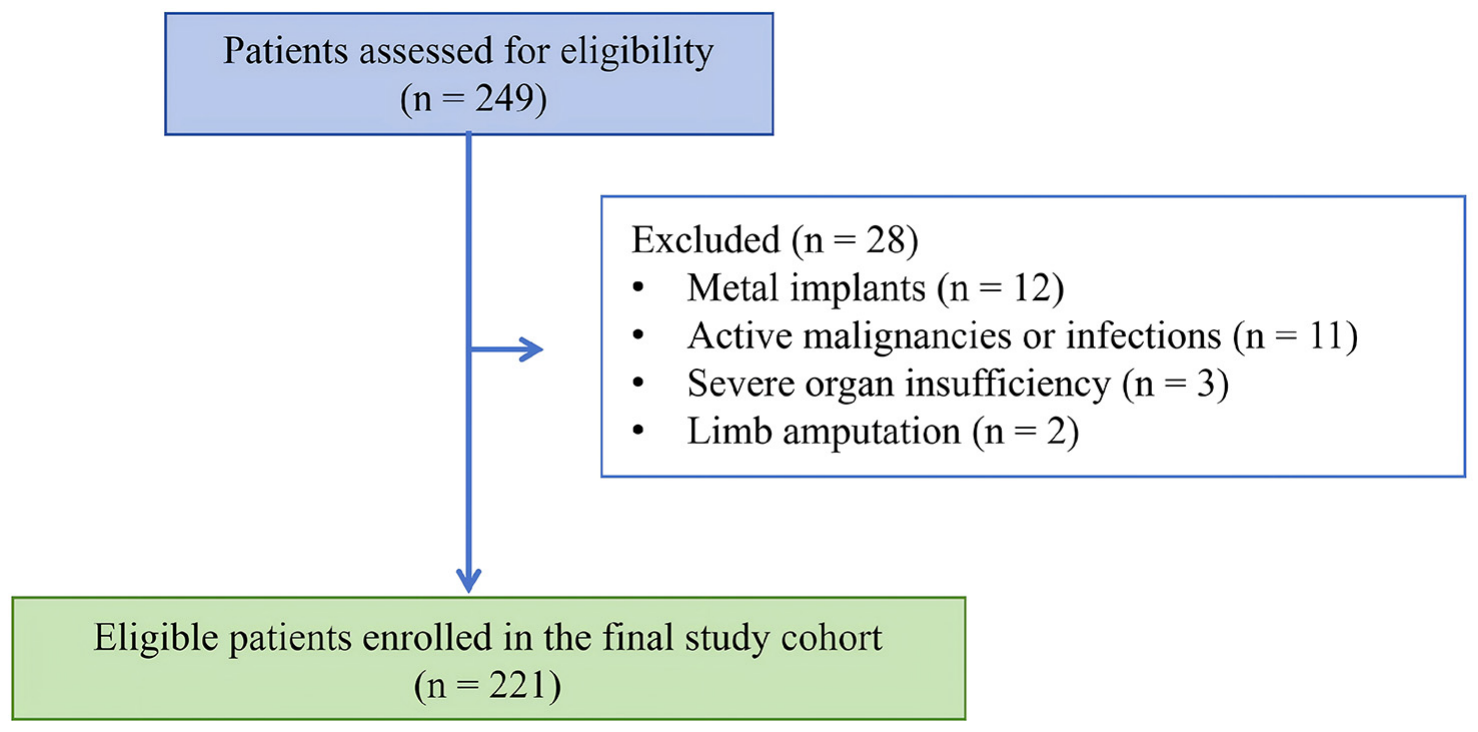
Flowchart of the study population.

Between January 1, 2024, and June 15, 2025, a total of 249 patients undergoing maintenance hemodialysis (MHD) at the Blood Purification Center of Wuxi People's Hospital were initially assessed for eligibility. The inclusion criteria were: (1) aged 18–75 years; and (2) receiving stable hemodialysis (thrice weekly for ≥3 months). Patients were excluded if they met any of the following criteria: (1) presence of metal implants; (2) history of limb amputation; (3) severe organ insufficiency; or (4) active malignancies or severe infectious diseases. Following screening, 28 patients were excluded (12 due to metal implants, 11 due to active malignancies or infections, 3 due to severe organ insufficiency, and 2 due to limb amputation). Ultimately, 221 eligible patients were enrolled in the final study cohort, which satisfied the pre-calculated sample size requirement (Figure 1).

### 2.2 Data collection

Baseline data were collected from all enrolled patients, covering the following domains: (1) sociodemographic characteristics, including age, sex, and marital status; (2) general clinical metrics, including height and dry weight, and dialysis vintage; and laboratory parameters, including serum levels of albumin, hemoglobin, calcium, phosphorus, and parathyroid hormone (PTH).

### 2.3 Muscle mass measurement

Body composition was assessed using the Fresenius Body Composition Monitor (model OP-ZHS 9/11.13, Fresenius Medical, Germany), a multifrequency bioelectrical impedance analyzer, specifically, immediately prior to the mid-week hemodialysis sessions to standardize the measurement timing. Following a 10-min rest in the supine position, whole-body bioimpedance spectroscopy was performed at 50 frequencies (5 kHz−1 MHz). Electrodes were placed on the distal upper and lower limbs of the non-fistula side. Body composition was then evaluated utilizing a three-compartment model to estimate excess fluid, lean tissue, and dry weight. Appendicular skeletal muscle mass (ASM) and the ASM index (ASMI) were calculated using Equations (1) and (2), respectively (18):


$$\begin{array}{l} ASM\left(kg\right)=0.276\times RI_{250kHz}+1.151\times sex+0.059\\ \;\times Xc_{5kHz}+0.429 \end{array}$$ (1)

$$\begin{array}{lll} ASMI\left({kg/m}^{2}\right) & = & \frac{ASM}{height^{2}} \end{array}$$ (2)

where RI represents the resistance index ($\frac{\Omega }{m^{2}}$); sex is defined as 1 for male and 0 for female; and Xc reactance represents reactance (Ω).

### 2.4 Handgrip strength measurement

As an indicator of muscle strength, handgrip strength was assessed prior to hemodialysis using a digital dynamometer. In accordance with the manufacturer's guidelines, measurements were obtained with the patient in a standing position, maintaining the elbow and forearm in a neutral posture. Testing was primarily conducted on the non-fistula hand; however, the dominant hand was utilized for patients with dialysis catheters. Two trials were performed, with each trial requiring a sustained grip for at least 3 s, and the maximum value was recorded for analysis.

### 2.5 Sarcopenia risk screening

Baseline sarcopenia risk was evaluated using the SARC-CalF questionnaire. The foundational SARC-F scale assesses five physical performance domains (strength, walking assistance, chair rising, stair climbing, and falls scored 0–10). Notably, a score of ≥4 has been shown to independently predict an elevated risk of sarcopenia in chronic kidney disease patients (19). To complement this functional assessment with an indicator of muscle mass, SARC-CalF incorporates calf circumference as a validated surrogate marker for skeletal muscle mass. By assigning an additional 10 points for a Calf circumference <34 cm (men) or <33 cm (women), a total score ≥11 (range 0–20) indicates a high sarcopenia risk. Compared with SARC-F alone, this integrated approach significantly improves screening sensitivity and overall diagnostic accuracy for probable sarcopenia (20).

### 2.6 Ultrasound measurements

**Figure 2:**
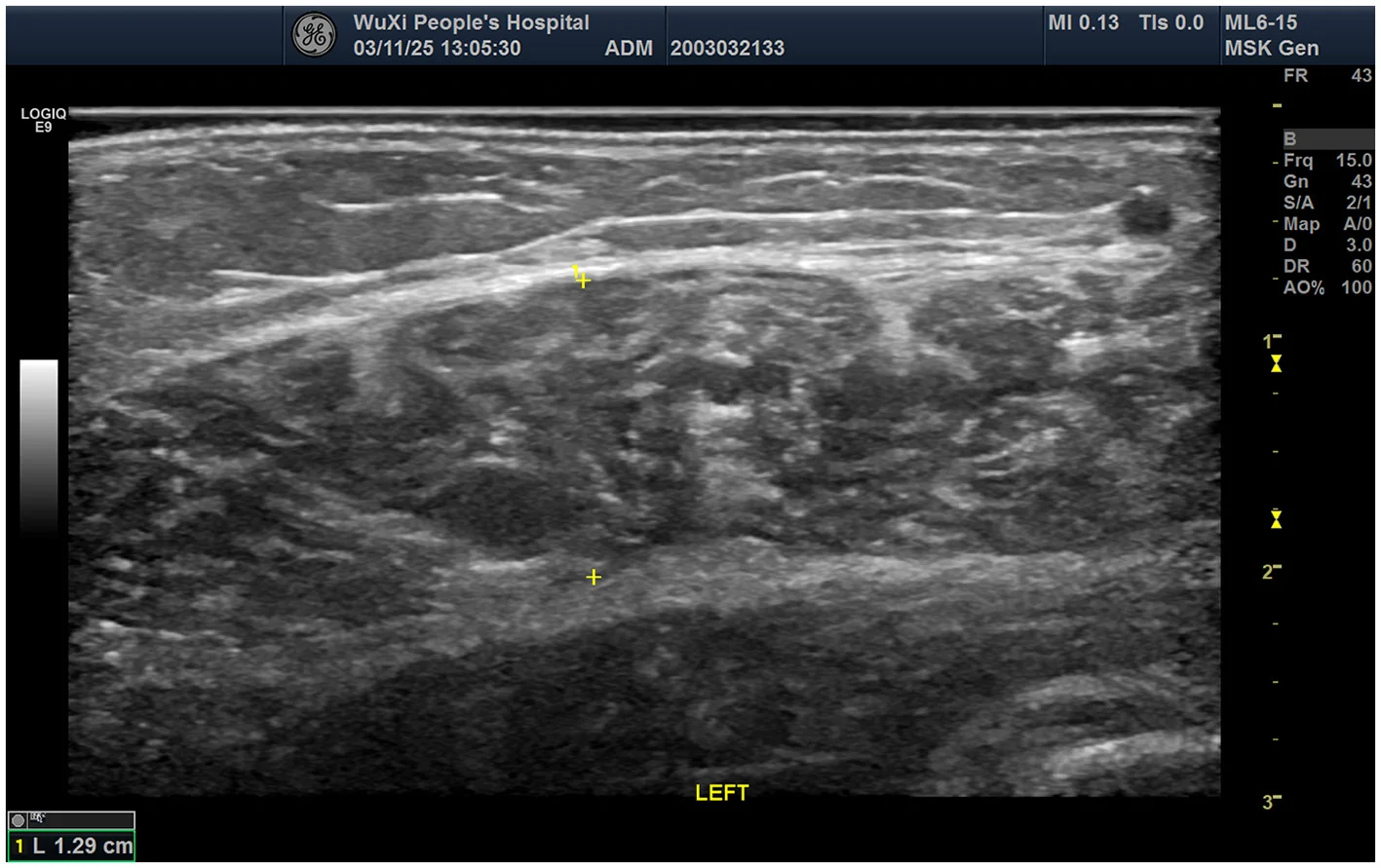
Measurement of medial gastrocnemius muscle thickness.

Skeletal muscle ultrasonography was performed prior to the hemodialysis session by a single experienced sonographer who had received standardized training in musculoskeletal ultrasound. Measurements were obtained using a GE Logiq E9 color Doppler system equipped with a high-frequency linear array transducer. Patients were placed in a semi-recumbent position with their knees flexed at 90° and feet resting flat. The measurement site for the medial gastrocnemius muscle was located at 30% of the tibial length (measured from the medial condyle of the tibia to the apex of the medial malleolus). To avoid any inadvertent compression of the superficial tissues and muscle architecture, a generous layer of ultrasound gel was applied, and the transducer was held lightly against the skin to ensure minimal compression. The MGMT and MGMPA were then measured at the midpoint of the muscle belly. Bilateral measurements were obtained, with each side measured twice, and the average values were calculated for the final analysis. A representative image of the MGMT measurement is shown in Figure 2.

### 2.7 Diagnostic criteria for sarcopenia

**Figure 3:**
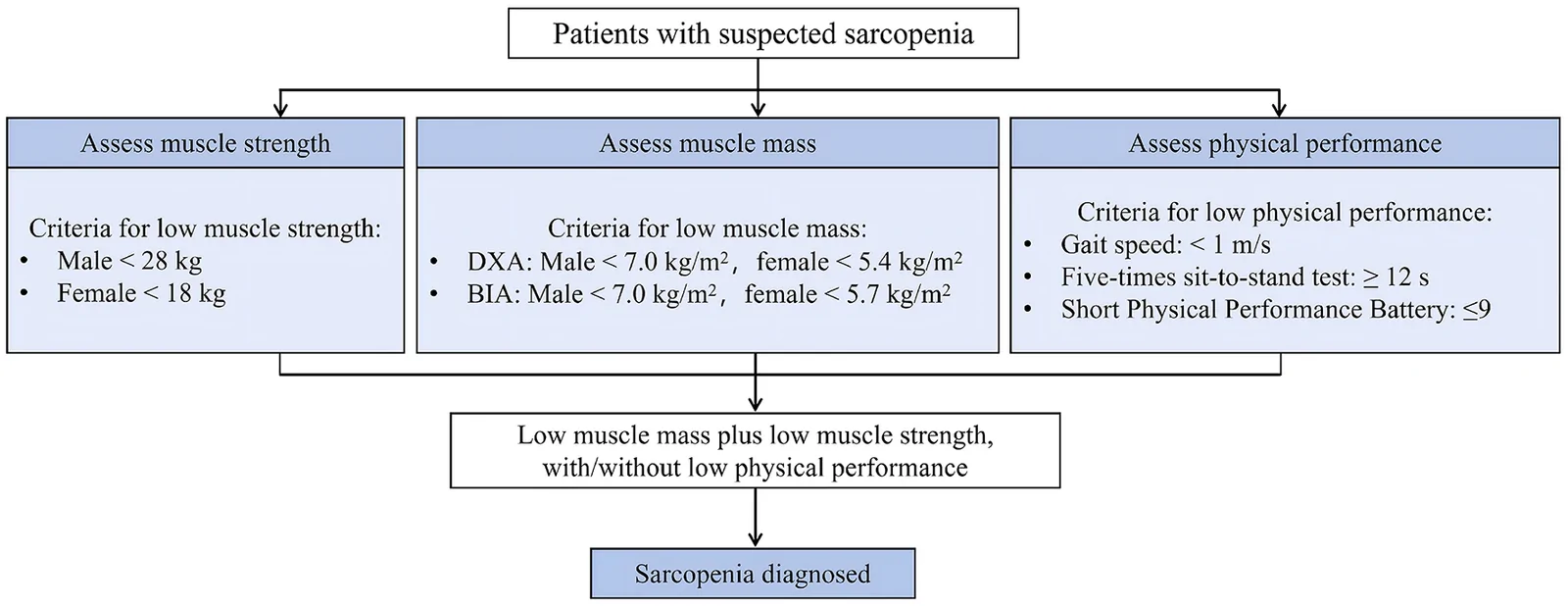
Sarcopenia diagnostic criteria.

In accordance with the AWGS 2019 guidelines, sarcopenia was diagnosed based on the concurrent presence of low muscle mass and low muscle strength, with or without reduced physical performance. The AWGS 2019 diagnostic criteria were utilized in this study to ensure methodological consistency across the entire cohort, as the clinical data compilation protocol was initiated prior to the publication of the updated AWGS 2025 consensus. The specific diagnostic cutoffs were defined as follows: (1) low muscle mass, indicated by a BIA-derived ASMI of <7.0 kg/m^2^ for men and <5.7 kg/m^2^ for women, and (2) low muscle strength, indicated by a handgrip strength <28 kg for men and <18 kg for women (Figure 3).

### 2.8 Data analysis

Continuous variables (assessed via the Shapiro-Wilk test) are expressed as mean ± standard deviation or medians (interquartile ranges), and were compared using the independent *t*-test or Mann–Whitney *U* test, respectively. Categorical variables are presented as frequencies (percentages) and analyzed using the chi-square test. Correlations were evaluated using Pearson's or Spearman's method. Diagnostic performance of ultrasonographic parameters was assessed using receiver operating characteristic (ROC) curve, with optimal cut-offs derived from the maximized Youden index. Multivariable logistic regression identified independent sarcopenia risk factors expressed as odds ratios [(ORs) and 95% CIs] to construct a diagnostic nomogram. The nomogram was evaluated via the area under the curve (AUC) for discrimination, calibration plots, and decision curve analysis (DCA) for clinical utility, followed by internal validation using 1,000 bootstrap resampling. Additionally, a bootstrap mediation analysis evaluated the direct effects of sonographic parameters on sarcopenia and their indirect effects mediated by age and albumin. Analyses were performed using SPSS25.0 and R 4.4.3. A two-sided *p* < 0.05 indicated statistical significance.

## 3 Results

### 3.1 Comparison of basic characteristics

The study cohort comprised 221 patients (140 males and 81 females). All participants underwent bioelectrical impedance analysis and ultrasonographic examination. The median age of the cohort was 54 years. Patients were stratified into non-sarcopenia (*n* = 171) and sarcopenia (*n* = 50) groups based on established cut-offs for ASMI and HGS; physical dysfunction was present in 10 patients (20%) in the sarcopenia group and 8 patients (4.7%) in the non-sarcopenia group, demonstrating a statistically significant difference (*p* = 0.002). The prevalence rate of sarcopenia was 22.6%.

The sarcopenia group was significantly older than the non-sarcopenia group (67 vs. 51 years, *p* < 0.001). However, the two groups showed no significant differences in gender (*p* = 0.202) or dialysis vintage (*p* = 0.240). Regarding laboratory parameters, no significant differences were observed in hemoglobin (*p* = 0.345), phosphorus (*p* = 0.073), and parathyroid hormone (*p* = 0.587), whereas serum albumin (38.25 vs. 40.8g/L, *p* = 0.048) and calcium (2.27 vs. 2.22 mmol/L, *p* = 0.028) showed statistical significance (Table 1).

**Table 1:** Patient characteristics: general, laboratory, nutritional, body composition, and ultrasound parameters.

Characteristics	Non-sarcopenia group (*n* = 171)	Sarcopenia group (*n* = 50)	*p*-value
Age (years)	51 (44,61)	67 (53,76)	**<0.001** ^***^
Gender			
Male	104 (60.8%)	36 (72.0%)	0.202
Female	67 (39.2%)	14 (28.0%)	
Dialysis vintage (years)	3(1,9)	6(2,9)	0.240
ASM (kg)	20.42 (17.30, 23.52)	18.33 (14.20, 19.60)	<0.001
ASMI (kg/m^2^)	7.25 (6.54, 8.07)	6.21 (5.53, 6.70)	<0.001
Lean tissue mass (kg)	41.09 ± 10.22	32.82 ± 8.25	<0.001
Physical Dysfunction	8 (4.7%)	10 (20%)	**0.002** ^**^
Albumin (g/L)	40.8(37.7, 42.0)	38.25(36.9, 40.9)	**0.048** ^*^
Hemoglobin (g/L)	110 (100,119).	112(101.3,122.8)	0.345
Calcium (mmol/L)	2.22 (2.07, 2.33)	2.27 (2.18, 2.40)	**0.028** ^*^
Phosphorus (mmol/L)	1.77 (1.44, 2.17)	1.58 (1.33, 1.95)	0.073
Parathyroid hormone (pg/mL)	221.8 (135.3, 345.6)	254.7 (155.6, 400.7)	0.587
Dry weight (kg)	65.6 (56.1, 72.7)	56.2 (51.1, 62.3)	**<0.001** ^***^
BMI (kg/m^2^)	23.6 (21.7, 26.6)	20.8 (19.0, 22.4)	**<0.001** ^***^
Grip strength (kg)	27.9 (21.2, 35.6)	16.9 (12.5, 20.0)	**<0.001** ^***^
Male	33.3 (28.0, 39.4)	19.1 (13.9, 23.0)	**<0.001** ^***^
Female	22.2 (18.9, 25.6)	14.5 (10.2, 16.1)	**<0.001** ^***^
Calf circumference (cm)	34.1 [32.0, 36.0]	30.0 [28.1, 32.0]	**<0.001** ^***^
Bioelectrical impedance indicators			
Overhydration (L)	1.2 (0.6, 2.2)	1.5 (0.9, 2.4)	0.348
Total Body Water (L)	35.6 (30.8, 40.5)	32.0 (25.8, 34.1)	**<0.001** ^***^
E/I	0.87 (0.79, 0.95)	0.92 (0.84, 1.04)	**0.006**
Ultrasound indicators			
MGMT (mm)	16.5 (14.9, 19.2)	12.8 (11.0, 14.7)	**<0.001** ^***^
Male	17.1 (15.4, 19.5)	13.5 (10.5, 14.7)	**<0.001** ^***^
Female	15.8 (14.6, 18.2)	12.7 (11.4, 13.4)	**<0.001** ^***^
MGMPA (°)	29.73 ± 5.99	26.35 ± 5.53	**<0.001** ^***^
Physical function			
SARC-calf score	0 (0, 10)	10 (10, 10)	**<0.001** ^***^

^*^p < 0.05, ^**^p < 0.01, ^***^p < 0.001. ASM, appendicular skeletal muscle mass; ASMI, appendicular skeletal muscle mass index; MGMT, medial gastrocnemius muscle thickness; MGMPA, medial gastrocnemius muscle pennation angle.

Patients with sarcopenia exhibited poorer nutritional status characterized by lower dry weight (56.2 vs. 65.6 kg, *p* < 0.001), BMI (20.8 vs. 23.6 kg/m^2^, *p* < 0.001), and calf circumference (30.0 vs. 34.1 cm, *p* < 0.001). Bioelectrical impedance analysis revealed that total body water was significantly lower (32.0 vs. 35.6 L, *p* < 0.001) and the E/I ratio was elevated (0.92 vs. 0.87, *p* = 0.006) in the sarcopenia group; however, overhydration did not differ significantly between the two groups. Ultrasonic indicators demonstrated a significant reduction in the sarcopenia group for both MGMT (12.8 vs. 16.5 mm) and MGMPA (26.35° vs. 29.73°) (both *p* < 0.001) (Table 1).

### 3.2 Ultrasonographic parameters correlate significantly with sarcopenia indicators

**Figure 4:**
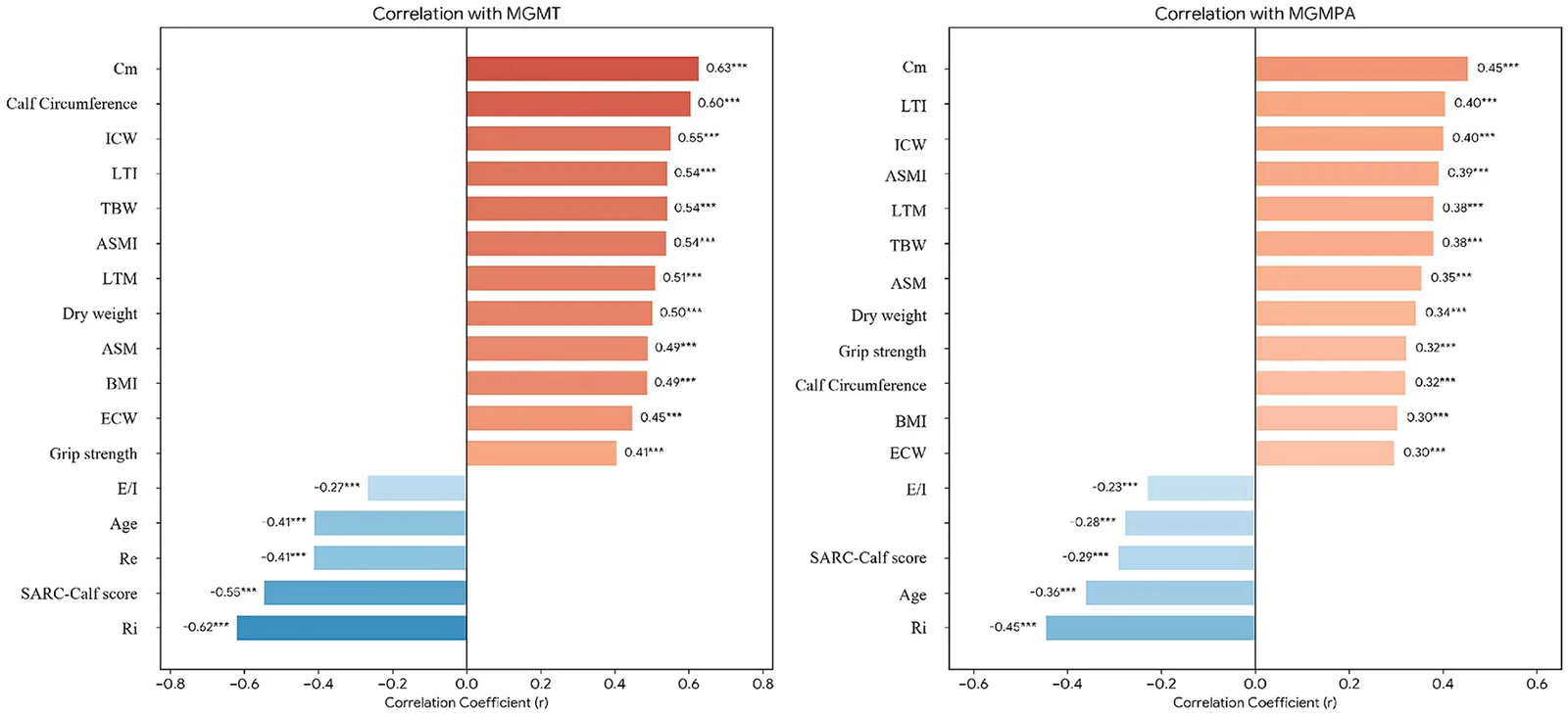
Correlations between ultrasonographic parameters and sarcopenia indicators. MGMT, medial gastrocnemius muscle thickness; MGMPA, medial gastrocnemius muscle pennation angle; ASMI: appendicular skeletal muscle mass index; ASM: appendicular skeletal muscle mass; BMI: body mass index. ***p < 0.001.

Spearman's rank correlation analyses demonstrated significant associations between ultrasonographic parameters, key sarcopenia indicators, and body composition metrics. These correlations are visually summarized in the diverging bar charts (Figure 4). Specifically, MGMT showed significant positive correlations with calf circumference (ρ = 0.600, *p* < 0.001), and bioimpedance-derived parameters such as ICW (ρ = 0.55, *p* < 0.001) and LTI (ρ = 0.54, *p* < 0.001). Furthermore, MGMT was significantly positively correlated with ASMI (ρ = 0.54, *p* < 0.001), ASM (ρ = 0.49, *p* < 0.001), BMI (ρ = 0.49, *p* < 0.001), and grip strength (ρ = 0.41, *p* < 0.001). Conversely, a

A similar trend was observed for MGMPA, which correlated positively with LTI (ρ = 0.40, *p* < 0.001), ICW (ρ = 0.40, *p* < 0.001), ASMI (ρ = 0.39, *p* < 0.001), ASM (ρ = 0.35, *p* < 0.001), grip strength (ρ = 0.32, *p* < 0.001), and calf circumference (ρ = 0.32, *p* < 0.001), while remaining negatively correlated with age (ρ = −0.36, *p* < 0.001) and the SARC-CalF score (ρ = −0.29, *p* < 0.001).

### 3.3 MGMT demonstrates good diagnostic performance for sarcopenia

**Figure 5:**
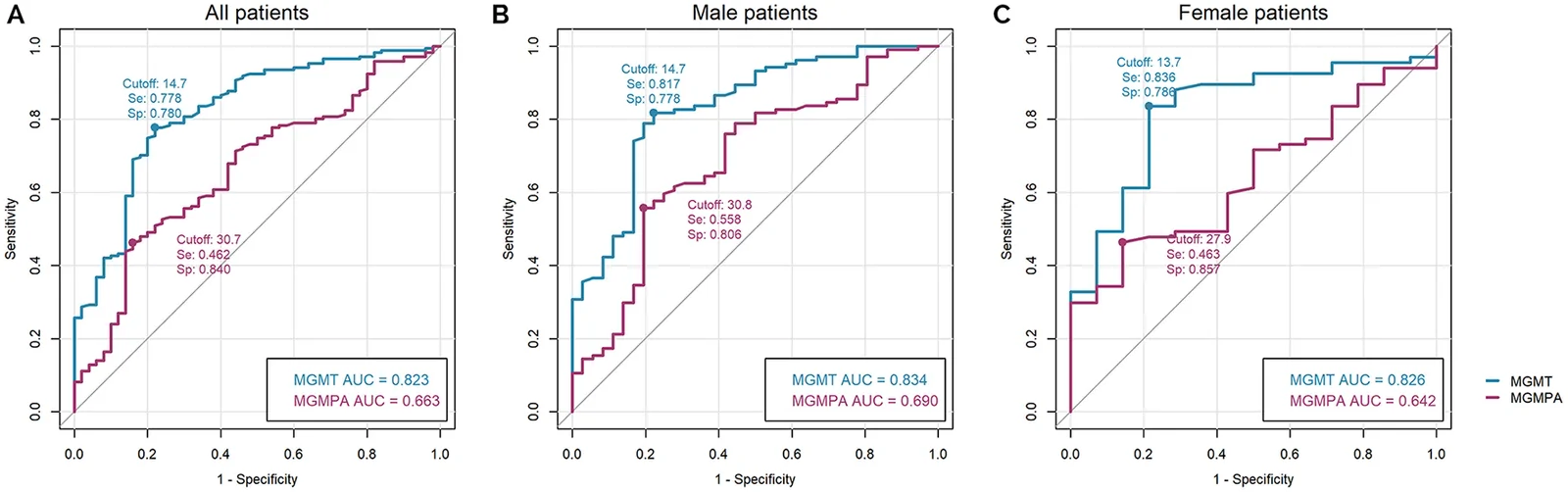
Diagnostic performance of GMT and GMPA for sarcopenia in different subgroups. **(A)** All participants, **(B)** Men, **(C)** Women. MGMT, medial gastrocnemius muscle thickness; MGMPA, medial gastrocnemius muscle pennation angle.

ROC curve analysis demonstrated that MGMT had a good diagnostic performance for sarcopenia, with an area under the curve (AUC) of 0.823 (95% CI: 0.756–0.889, *p* < 0.001). The optimal cut-off value of 14.7 mm was determined using the Youden index, yielding a sensitivity of 0.778 and a specificity of 0.780. After sex stratification, the diagnostic performance of MGMT remained robust. In males, the AUC was 0.834 (95% CI: 0.756–0.912, *p* < 0.001) with an optimal cut-off value of 14.7 mm (sensitivity, 0.817; specificity, 0.778). In females, the AUC was 0.826 (95% CI: 0.707–0.944, *p* < 0.001), with an optimal cut-off of 13.7 mm (sensitivity, 0.836; specificity, 0.786) (Table 2; Figures 5A–C).

**Table 2:** ROC curve analysis for sarcopenia diagnosis by MGMT and MGMPA.

Parameters	AUC	*p*-value	Cutoff value (mm)	Sensitivity	Specificity
MGMT (mm)					
all patients	0.823	<0.001^***^	14.7	0.778	0.780
Male	0.834	<0.001^***^	14.7	0.817	0.778
female	0.826	<0.001^***^	13.7	0.836	0.786
MGMPA (°)					
All patients	0.663	0.003^**^	30.7	0.462	0.840
Male	0.690	0.015	30.8	0.558	0.806
female	0.642	0.048	27.9	0.463	0.857

^**^p < 0.01, ^***^p < 0.001. MGMT, medial gastrocnemius muscle thickness; MGMPA, medial gastrocnemius muscle pennation angle.

Additionally, the diagnostic utility of MGMPA was evaluated. While statistically significant, MGMPA exhibited a comparatively lower diagnostic performance, with an AUC of 0.663 (*p* = 0.003) and a cut-off value of 30.7° (sensitivity, 0.462; specificity, 0.840) in all patients. Similar trends were observed in male (AUC = 0.690, *p* = 0.015) and female (AUC = 0.642, *p* = 0.048) subgroups.

### 3.4 Multivariable analysis identifies independent factors associated with sarcopenia

**Figure 6:**
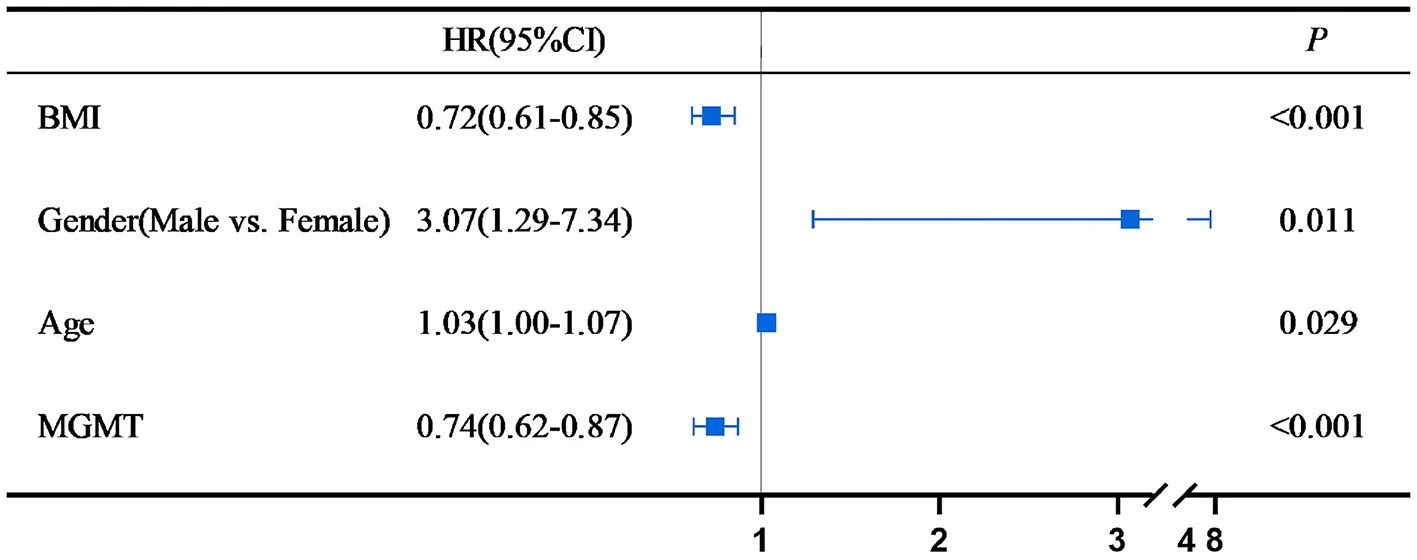
Forest plot for multivariable logistic regression analysis of factors associated with sarcopenia. The model was adjusted for age, gender, diabetes, dry weight, MGMPA, and calf circumference. Odds ratio (OR) and 95% confidence interval (CI) are shown. MGMT, medial gastrocnemius muscle thickness; MGMPA, medial gastrocnemius muscle pennation angle; BMI, body mass index.

As shown in Figure 6, a multivariable logistic regression analysis was performed to determine the independent predictors of sarcopenia in MHD patients. The model included BMI, gender, age, and MGMT. The results revealed that MGMT [Odds Ratio (OR) = 0.74, 95% CI: 0.62–0.87, *p* < 0.001] and BMI [OR = 0.72, 95% CI: 0.61–0.85, *p* < 0.001] were significant independent protective factors, where higher values were associated with a lower risk of sarcopenia. Conversely, male gender [OR = 3.07, 95% CI: 1.29–7.34, *p* = 0.011] and older age [OR = 1.03, 95% CI: 1.00–1.07, *p* = 0.029] were identified as independent risk factors.

**Figure 7:**
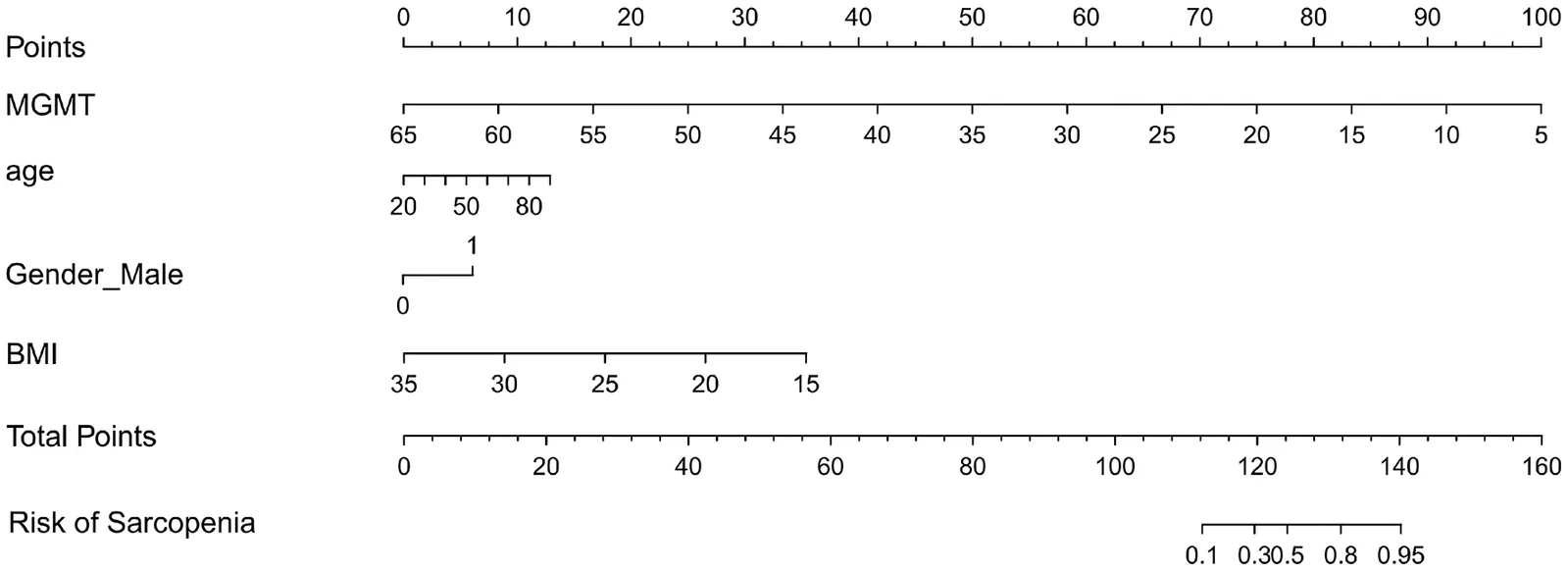
Nomogram for predicting the risk of sarcopenia. MGMT, medial gastrocnemius muscle thickness; BMI, body mass index.

**Figure 8:**
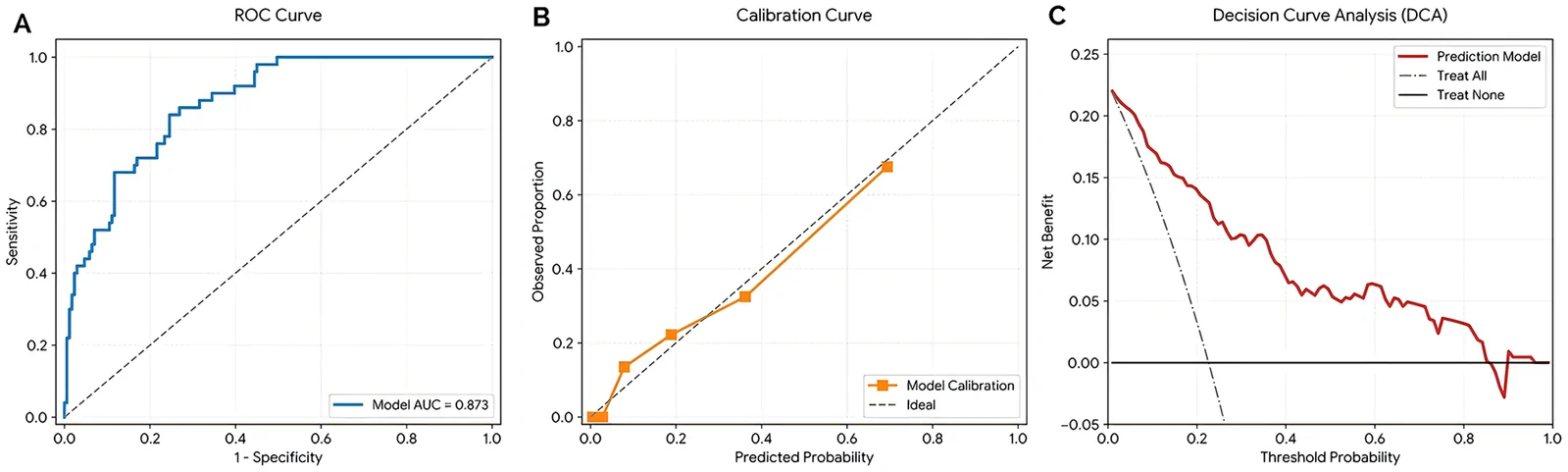
Verification of nomogram for predicting the risk of sarcopenia. **(A)** ROC curve of nomogram (AUC = 0.873, 95% CI: 0.824–0.922). **(B)** Calibration curve of nomogram. **(C)** DCA curve of nomogram. ROC, receiver operating characteristic; DCA, decision curve analysis; AUC, area under the curve.

The identified independent predictors (MGMT, BMI, age, and gender) were incorporated to construct a nomogram for predicting sarcopenia risk (Figure 7). Discrimination analysis revealed that the area under the ROC curve of the nomogram was 0.873 (95% CI: 0.824–0.922) (Figure 8A). Furthermore, following 1,000 bootstrap internal validation resamplings, the optimism-corrected AUC was 0.860, demonstrating that the risk of model overfitting was reduced and within a controllable range. The slope of the calibration curve was close to 1, suggesting that the model had high accuracy (Figure 8B). The DCA showed that the model had potential clinical decision-making value across a wide range of threshold probabilities (Figure 8C).

### 3.5 Direct association between MGMT and the presence of sarcopenia revealed by mediation analysis

**Figure 9:**
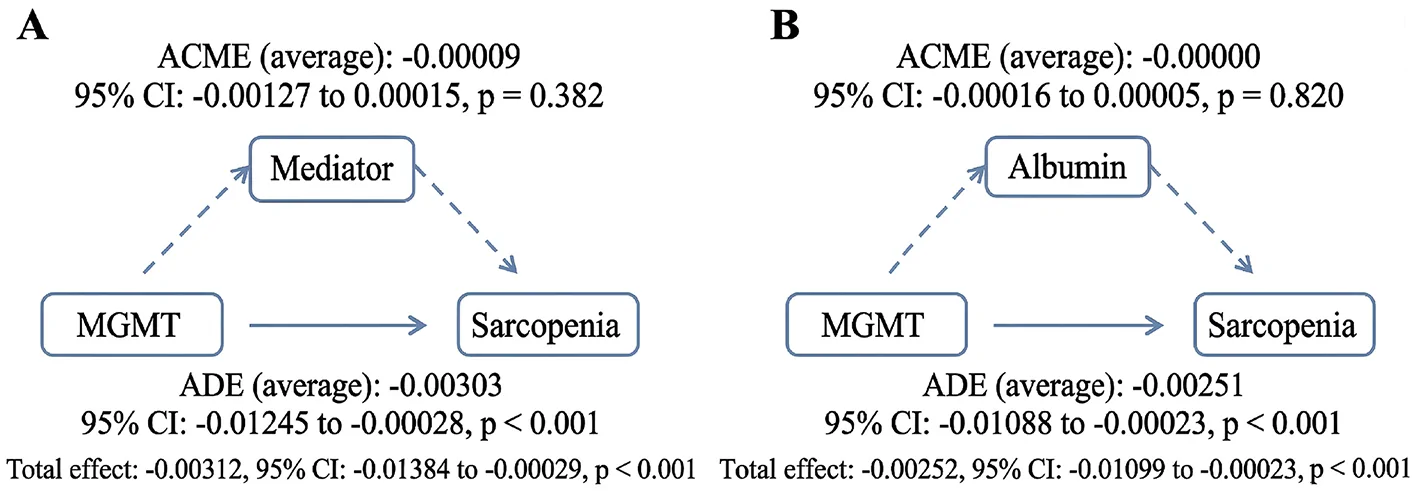
Mediation analysis of the potential pathways associated with MGMT and the presence of sarcopenia. The association of MGMT with the presence of sarcopenia directly and indirectly through relationships mediated by albumin **(A)** and age **(B)** were analyzed. ACME, average causal mediation effect; ADE, average direct effect; MGMT, medial gastrocnemius muscle thickness.

In our analysis, we assessed the potential mediating roles of age and albumin in the relationship between MGMT and sarcopenia. Mediation analysis revealed no significant indirect (mediating) effects through age (ACME: −0.00009, 95% CI: −0.00127–0.00015, *p* = 0.382) or albumin (ACME: −0.00000, 95% CI: −0.00016–0.00005, *p* = 0.820) (Figures 9A, B). Conversely, MGMT demonstrated a significant direct association with the presence of sarcopenia in both models (age model ADE: −0.00303, 95% CI: −0.01245 to −0.00028, *p* < 0.001; albumin model ADE: −0.00251, 95% CI: −0.01088 to −0.00023, *p* < 0.001). These findings indicate that the association between MGMT and sarcopenia exists independently of age and serum albumin levels. All analyses were adjusted for dry weight, BMI, and calf circumference.

## 4 Discussion

This study aimed to investigate the feasibility and diagnostic performance of ultrasonographic MGMT and MGMPA for assessing sarcopenia in patients undergoing MHD. The results demonstrated a significant correlation between the MGMT and bioelectrical impedance analysis-derived ASMI, along with a general reduction in ASM within this population.

Previous studies have found the clinical significance of gastrocnemius muscle mass in non-MHD populations. A meta-analysis demonstrated that muscle thickness measurements of the gastrocnemius, rectus femoris, and tibialis anterior muscles exhibited moderate diagnostic accuracy for sarcopenia (21). Because the gastrocnemius muscle plays an integral role in daily functional activities and its thickness strongly correlates with whole-body muscle mass, its superficial location makes it ideal target for accessible ultrasonographic measurement (17, 22). For instance, Yuguchi et al. (23) reported a thickness cut-off of <11.6 mm in 195 older Japanese adults, While Jing et al. (24) proposed 1.50 cm for older Chinese adults. Furthermore, a study of 269 Japanese community-dwelling adults reported sex-stratified cut-offs of 1.53 cm for men and 1.42 cm for women (25).

Despite these established findings in general aging populations, evidence evaluating the clinical utility of gastrocnemius muscle thickness specifically in MHD patients remains relatively scarce. While Tao et al. (17) replicated similar cut-off values (1.53 cm for men, 1.41 cm for women) in an MHD cohort, comprehensive studies exploring its diagnostic performance and its detailed correlation with body composition indicators in this highly vulnerable population are still lacking. To address this gap, the present study systematically evaluated the diagnostic performance of ultrasonographic MGMT for sarcopenia in patients on maintenance hemodialysis. Our analysis revealed that MGMT is significantly associated with key sarcopenia-related indicators, thereby providing preliminary, sex-stratified evidence regarding its potential value as an accessible surrogate screening tool in the MHD setting.

In this study, MGMPA was significantly reduced in the sarcopenia group and demonstrated significant correlations with key sarcopenia-related indicators, including ASMI, BMI, handgrip strength, and calf circumference. However, ROC curve analysis revealed that while statistically significant, MGMPA exhibited a comparatively lower diagnostic performance (AUC = 0.663) than MGMT. This suggests that although the pennation angle undergoes structural alterations during muscle wasting, it may lack sufficient standalone discriminative power in this cohort. The pennation angle represents the angle between muscle fascicles and the deep aponeurosis, determining the muscle's mechanical efficiency in force transmission through the fascial plane. In patients with low muscle mass, a lower pennation angle is often observed alongside a reduction in gastrocnemius muscle thickness; however, these interrelated structural alterations may mask straightforward intergroup differences in a cross-sectional setting, thereby limiting the standalone diagnostic utility of MGMPA (16). Nonetheless, several studies have combined gastrocnemius muscle thickness with other ultrasonographic parameters, such as cross-sectional area and pennation angle, for sarcopenia diagnosis, demonstrating high diagnostic performance (26).

Sarcopenia is a multifaceted syndrome driven by muscle mass depletion, abnormal nutritional status, metabolic derangements, and systemic factors; therefore, relying on a single structural indicator may not comprehensively capture the disease state. The diagnostic accuracy of various ultrasound parameters—such as muscle thickness, cross-sectional area, pennation angle, and echogenicity—varies significantly depending on the study population (27). In this context, MGMT primarily captures macroscopic declines in muscle volume, which closely aligns with the core pathophysiological loss of muscle mass in sarcopenia. In contrast, MGMPA is more indicative of microscopic muscle fiber arrangement and structural mechanics (12). Although MGMPA correlated with several sarcopenia-related indices in our cohort, its overall diagnostic performance was markedly lower than that of MGMT. This discrepancy is likely exacerbated by the unique pathophysiological milieu of dialysis patients, where profound fluid shifts and intramuscular fat infiltration can severely confound the isolated measurement of the pennation angle (28). Consequently, given its robust and independent predictive capability, only MGMT was incorporated into our final diagnostic nomogram.

This study demonstrated that ultrasonographic MGMT is a potentially reliable tool for assessing sarcopenia in patients undergoing MHD, exhibiting acceptable diagnostic performance, and offering a practical alternative for routine clinical screening in this population. However, owing to practical constraints of this study, muscle mass was evaluated using BIA instead of DXA, the latter being the gold standard and minimally affected by hydration status. Although BIA measurements were taken immediately before the mid-week dialysis session, changes in volume status, particularly interdialytic weight gain and volume load, can significantly affect BIA measurement results. Fluid retention leads to an overestimation of extracellular water, which inevitably confounds the accuracy of BIA-estimated lean tissue and appendicular skeletal muscle mass. This potential interference from unquantified volume load remains a limitation and may have affected the exact precision of our study results. Furthermore, the accuracy of the BIA-derived estimates depends heavily on the specific formulas used. The ASM estimation equations applied here were originally developed for Caucasian MHD population and require further external validation in Asian cohorts. Lastly, individual fluctuations in hydration status remain an inherent challenge in interpreting BIA data within this clinical setting (29).

In recent years, numerous studies have highlighted the clinical value of ultrasonography for evaluating muscle quality. Its unique advantage lies in the ability of high-resolution probes to visualize key morphological alterations, including changes in muscle echogenicity, the expansion of hypoechoic areas between muscle bundles, and variations in muscle volume. These sonographic features serve as important indicators reflecting intramuscular fat infiltration and a reduction in muscle mass. However, several limitations of ultrasonography in sarcopenia assessment should be acknowledged. For instance, the metrics derived from a single lower-extremity muscle may not adequately reflect whole-body muscle status. Importantly, sarcopenia has been independently associated with an increased risk of cardiovascular events as well as a higher susceptibility to falls and fractures in this vulnerable population. Given the cross-sectional nature of the current evidence, future longitudinal studies are needed to further evaluate the predictive value of these ultrasonographic parameters for long-term clinical outcomes.

This study explored the potential exploratory mediation relationship between MGMT and sarcopenia. The results indicated that although age and albumin are theoretically considered core risk factors for sarcopenia, they did not exhibit significant mediating effects in the statistical association between MGMT and sarcopenia. This suggests that association between MGMT and sarcopenia may involve other complex factors independent of the aging process and nutritional status. Sarcopenia in the MHD population is widely recognized as a multi-factorial condition involving uremic toxins, dialysis-induced catabolism, and a hypermetabolic state (30, 31); these interrelated pathological factors likely form the background against which the prominent statistical correlation between MGMT and sarcopenia is observed. Importantly, as a macroscopic morphological parameter, variations in MGMT are more appropriately conceptualized as a downstream manifestation of intrinsic pathophysiological processes rather than an initial driver of the condition. Consequently, relying solely on this single structural indicator may have inherent limitations in capturing subtle or multi-dimensional skeletal muscle alterations. Therefore, while our findings support the diagnostic performance of MGMT, future research should explore the clinical value of integrating structural ultrasonography with molecular or functional biomarkers to achieve a more comprehensive assessment of sarcopenia in MHD patients (31–34).

Our study has several limitations that should be acknowledged. First, the ASM formulas applied in this study were derived from non-Asian populations, and their generalizability to Asian cohorts requires further external verification. Second, performing BIA shortly before dialysis introduces potential confounding effects related to hydration status. Third, assessments based on a single-muscle site may not fully reflect whole-body muscle mass. Fourth, the predictive nomogram developed in this study relies on internal validation, and future external validation across multi-center cohorts is necessary. Furthermore, it is important to note that Decision Curve Analysis (DCA) is primarily used to evaluate the theoretical net benefit of a predictive model at varying risk thresholds. Our DCA results only suggest a potential clinical decision-making value and cannot directly prove that the model improves actual patient clinical outcomes. Fifth, although all ultrasound evaluations were performed by a single experienced sonographer to minimize inter-observer variability, a formal intra-operator reproducibility analysis was not conducted, which remains a methodological limitation. Consequently, future longitudinal studies are needed to investigate the long-term predictive value of these ultrasonographic parameters for adverse clinical outcomes.

In conclusion, MGMT serves as a practical and non-invasive tool that is significantly associated with sarcopenia in patients undergoing MHD, demonstrating acceptable cross-sectional diagnostic performance. This approach offers a valuable option for routine clinical screening, although its longitudinal utility for risk stratification and its direct impact on patient prognosis warrant further multi-center investigation.

## Data Availability

The raw data supporting the conclusions of this article will be made available by the authors, without undue reservation.
